# Implications of Hyponatremia in Liver Transplantation

**DOI:** 10.3390/jcm4010066

**Published:** 2014-12-29

**Authors:** Sertac Cimen, Sanem Guler, Subhashini Ayloo, Michele Molinari

**Affiliations:** Department of Surgery and Community Health, Dalhousie University, 1276 South Park Street, Halifax, B3H 2Y9, NS, Canada; E-Mails: sertaccimen@yahoo.com (S.C.); gulersanem@yahoo.com (S.G.); drsayloo@gmail.com (S.A.)

**Keywords:** hyponatremia, liver transplantation, sodium, cirrhosis

## Abstract

Although there are a limited number of quality studies, appropriate peri-operative management of serum electrolytes seems to reduce adverse outcomes in liver transplantation. Hyponatremia is defined as the presence of serum concentration of sodium equal ≤130 mmol/L and it is detected in approximately 20% of patients with end stage liver disease waiting for a liver transplant (LT). This paper will focus on the pathogenesis of dilutional hyponatremia and its significance in terms of both candidacy for LT and post-operative outcomes.

## 1. Pathogenesis of Hyponatremia in Cirrhotic Patients

**Figure 1:**
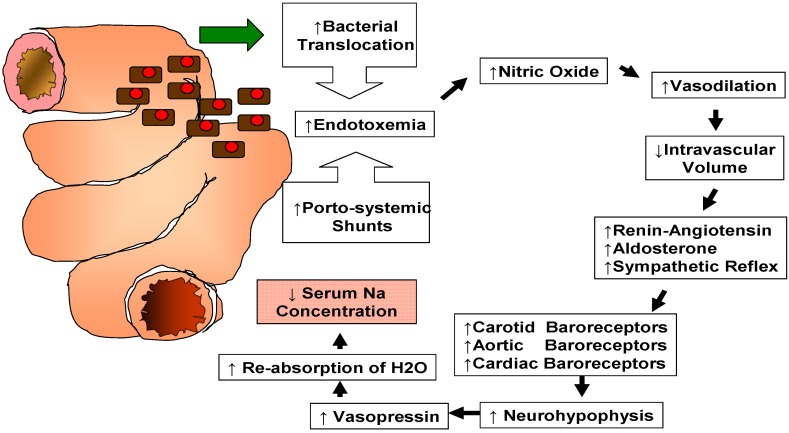
Pathophysiology of dilutional hyponatremia in patients affected by end stage liver disease.

A variety of factors can contribute to the development of hyponatremia in patients with end stage liver disease [1]. Its mechanism is complex, reflecting a generalized hemodynamic derangement as illustrated in Figure 1.

Recent studies have shown that increased intestinal bacterial translocation combined with portosystemic shunting leads to endotoxemia and increased production of endothelial nitric oxide causing systemic and splanchnic vasodilation [1]. Systemic vasodilation leads to a reduction of the effective circulating volume with stimulation of the renin-angiotensin-aldosterone system (RAAS) [2]. Stimulation of RAAS and decreased hepatic clearance of aldosterone result in hyperaldosteronism [3]. On the other hand, systemic vasodilation stimulates a sympathetic nervous response [2]. Carotid, cardiac and aortic arch baroreceptors activate the signaling pathway of the neurohypophysis with secretion of arginine vasopressin (AVP). AVP acts via V2 receptors on the renal collecting tubules triggering movement of aquaporin-2 water channels to the apical membranes causing re-absorption of water [1]. These changes altogether lead to a decrease in serum sodium concentration despite the existence of increased renal sodium reabsorption and high total body sodium content [2]. Therefore, this type of hyponatremia is referred to as “dilutional” hyponatremia. Reduction in the delivery of filtrate to the distal nephron and decreased renal prostaglandin synthesis are also known to participate in the impairment of solute-free water excretion in cirrhotic patients with subsequent development of dilutional hyponatremia [2].

## 2. Clinical Implications

In 1956, Dame Sheila Sherlock observed that “In patients with liver disease, serum-Na levels below 130 mEq/L must be regarded as serious, and if below 125 mEq/L ominuous” [4].

In a large international cooperative survey of cirrhotic patients, Angeli *et al.* [5] found that hyponatremia was associated with greater frequency of refractory ascites as well as hepatic encephalopathy, spontaneous bacterial peritonitis and hepatorenal syndrome (HRS). Similarly, Gines *et al.* [6] found that hyponatremia was an independent predictor of HRS and Guevara *et al.* [7] reported that hyponatremia was a major risk factor for the development of hepatic encephalopathy. Borroni *et al.* [8] showed that in-hospital mortality was significantly higher in cirrhotic patients with hyponatremia compared to those with normal sodium level (26% *vs.* 9%). In addition, these authors found that the subgroup with serum sodium ≤125 mEq/L had the highest risk of death (48%).

The negative impact of hyponatremia is well known but it is not very clear whether this is due to the fact that it might be a marker of the stage of the disease or if that is due to the negative effects of low serum sodium levels on the function of several organs (*i.e.*, central nervous system, circulatory system).

## 3. Predicting Mortality in Cirrhotic Patients Waiting for Liver Transplantation

The Model for End Stage Liver Disease (MELD) is a scoring system used to measure the severity of chronic liver disease [9]. Patient’s serum bilirubin, creatinine and international normalized ratio for prothrombin time (INR) are entered in a logistic regression probability function predicting the mortality risk of cirrhotic patients within a 3 months period [10].

It was initially developed for prediction of mortality in patients who had undergone a transjugular intrahepatic portosystemic shunt (TIPS) procedure [9]. It was subsequently found to be useful in prioritizing the allocation of liver grafts [10,11,12].

The MELD score is not a perfect model and many investigators have worked on improving its accuracy [9,13,14]. One of these efforts focused on incorporating the serum sodium level into the MELD model which led to a revised model called “MELD-Na” [14]. This effort was supported by several studies that found a positive correlation between hyponatremia and preoperative mortality in LT candidates [15,16]. Heuman *et al.* [15] reported that hyponatremia was a strongly predictive variable of short-term mortality independently from the MELD score. Ruf *et al.* [16] supported those findings in 262 waitlisted cirrhotic patients where hyponatremia was present in 63% of patients who died compared to 13% of those who survived. Their results also suggested that serum sodium was an earlier and more sensitive test than serum creatinine to identify circulatory dysfunction that could lead to renal failure and death.

Despite these findings, serum sodium concentration may decrease with fluid overload and with the use of diuretics and therefore it might be manipulated causing some advantage for selected patients waiting for a LT [16]. Hence, the United Network of Organ Sharing (UNOS) had reservations regarding a complete switch from MELD formula to MELD-Na model to prevent this issue [13,14,16,17,18,19].

## 4. Pretransplant Management of Hyponatremia

Determination of the patient’s volume status is the first step in the management of patients affected by hyponatremia [20]. As illustrated in Figure 1, the vast majority of patients with advanced cirrhosis suffer from hypervolemic (dilutional) hyponatremia [21]. Only a small subset of patients are affected by hyponatremia due to diuretics [20,22] that can be corrected by decreasing their dose or by replacing intravascular volume with solutions rich in sodium [20,22].

**Figure 2:**
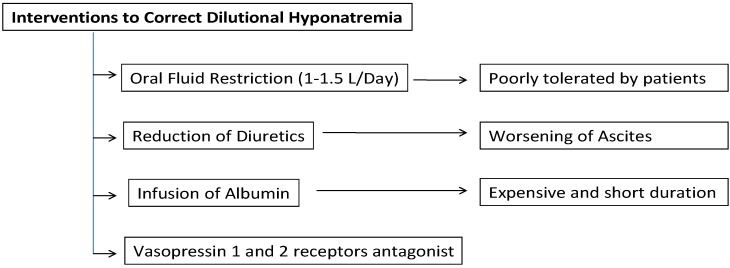
Interventions currently available to correct dilutional hyponatremia in cirrhotic patients.

To date (Figure 2), the mainstay of pretransplant management of “hypervolemic” hyponatremia has revolved around fluid restriction (1–1.5 L/day) and reduction of diuretics. However, fluid restriction is usually not well tolerated by these patients and the interruption of diuretics often leads to tense ascites [22]. Parenteral infusion of albumin is effective in restoring a more physiological circulatory volume and therefore it is useful in patients with severe hyponatremia. However, it is expensive and the duration of its positive effects is relatively short [1]. Another common treatment option is sodium bicarbonate [23]. Nevertheless, its high sodium concentration (1000 mEq/L) can be problematic since it might cause central pontine myelinolysis (CPM). It can also lead to myocardial depression due to worsened intracellular acidosis, especially with copious and/or rapid administration. Therefore, in the clinical settings where copious administration of sodium bicarbonate is anticipated, tris-hydroxymethyl aminomethane (THAM) is considered as a reasonable alternative. Since THAM is a weak base, it does not exacerbate intracellular acidosis and consequent myocardial depression. However, it has much lower sodium content than sodium bicarbonate and thus, it cannot completely negate the use of sodium bicarbonate particularly in severely hyponatremic patients [24].

A new class of pharmacological agents, the “vaptans” (*i.e.*, conivaptan, tolvaptan, lixivaptan) is showing promising results [25]. They selectively block the vasopressin receptors in the cells of the renal collecting system causing a significant increase in solute-free water excretion. Their use allows avoidance of fluid restriction and the continuation of natriuretic diuretics.

O’Leary *et al.* [26] treated 24 patients with cirrhosis and hyponatremia with intravenous vasopressin-1 and vasopressin-2 receptors antagonist conivaptan. Serum sodium concentration could be increased safely in the majority of these patients and 60% to 67% had a 5 mmol/L increase in serum sodium. Patients who subsequently underwent LT did well and none had neurological sequelae associated with hyponatremia.

Although vaptans seem to be safe and effective for the management of difficult cases of hyponatremia, they are still not widely available in patients with end stage liver disease [26,27]. Moreover, it is still unclear whether the correction of serum sodium levels will improve the overall post-OLT outcomes.

## 5. Impact of Hyponatremia on Post-Transplant Outcomes

Although hyponatremia is associated with reduced survival in patients with cirrhosis awaiting LT, it remains unclear whether hyponatremia itself represented a risk factor for adverse outcomes after LT.

A handful of previous relatively small observational studies had demonstrated a correlation between recipient hyponatremia and post-transplant mortality [28,29,30]. However, an equivalent number of similar quality studies have challenged this notion [31,32,33].

Hackworth *et al.* [1] performed a retrospective analysis of 213 patients and concluded that pretransplant hyponatremia was associated with prolonged duration of mechanical ventilation, ICU stays and hospitalization. In line with Hackworth *et al.* [1], Karapanagiotou *et al.* [34] showed that recipients who had pretransplant hyponatremia had higher rates of neurological complications, renal failure, mechanical ventilation requirements and prolonged ICU stay.

Londono *et al.* [2] evaluated the influence of recipient hyponatremia on survival at 3 months in a series of 241 consecutive LT patients. In this study, hyponatremic recipients had a higher rate of neurologic disorders, infectious complications and renal failure during the first month (odds ratio; 4.6, 3.4 and 2.7 respectively). More importantly, their 3-month survival after LT was significantly reduced (84% *vs.* 95% respectively; *p* < 0.05). Similar results were reported by Boin *et al.* [35] who analyzed 318 consecutive OLT recipients and concluded that pretransplant hyponatremia was associated with increased mortality. On the other hand, Kim *et al.* [32] and Cywinski *et al.* [33] found that preoperative hyponatremia did not predict postoperative mortality either at 3 or 6 months following LT.

In a large multicentric study of 5150 LTs by Dawwas *et al.* [36], serum sodium ≤130 mEq/L was associated with a 55% increase in 3-month perioperative mortality. In contrast, Yun *et al.* [31] reported that in 2175 LT recipients preoperative serum sodium had no impact on patients’ 3-month survival.

Fukuhara *et al.* [37] evaluated the impact of pretransplant hyponatremia on postoperative clinical outcomes in the setting of living donor liver transplantation (LDLT). They showed that pretransplant hyponatremia was a significant risk factor for short-term graft loss and postoperative complications including sepsis, renal failure and neurological disorders. In addition, serum sodium level was a significant independent predictive factor for post-transplant short-term graft loss.

## 6. Pathophysiology of Adverse Events Associated with Hyponatremia

It is known that dilutional hyponatremia in the setting of cirrhosis develops quite slowly [20,22]. The central nervous system adapts to the low osmolality by losing intracellular solutes such as inorganic ions and organic osmolytes [20,22]. When hyponatremia is corrected too rapidly, lack of adaptation by the brain may lead to a condition referred to as “osmotic demyelination syndrome” [20,22]. This syndrome is often associated with significant neurological morbidity and mortality and may present as CPM [20,22].

CPM is the most severe neurologic disorder described in hyponatremic patients [31]. Lee *et al.* [38] reviewed 1247 patients undergoing LT and documented 11 (0.88%) CPM cases. Londono *et al.* [2] and Dawwas *et al.* [36] suggested that correcting the serum sodium prior to transplantation may prevent the development of CPM as it will decrease the likelihood of a precipitous rise in serum sodium levels after LT.

Yun *et al.* [31] showed that the incidence of CPM was correlated with the presence of pretransplant hyponatremia. However, not all severely hyponatremic patients included in this study developed post-LT CPM and not all patients with post-LT CPM had preoperative low serum sodium levels. Hence, they suggested that a better understanding of the pathogenesis, risk factors and prevention policy of post-LT CPM was needed.

An alternative hypothesis proposed by Hackworth *et al.* [1] regarding the association of hyponatremia with post-LT adverse outcomes considers hyponatremia as a surrogate marker for other serious systemic abnormalities caused by end stage liver disease such as hypotension, malnutrition, poor performance status, impaired wound healing and depressed immune capacity.

## 7. Conclusions

Today, it is generally agreed that preventing and correcting severe hyponatremia is an important aspect of the pretransplant management of cirrhotic patients as it decreases their mortality risk while waiting for LT. However, the extent to which hyponatremia should be corrected to prevent post- transplant adverse events remains open to speculations as the results of current studies are conflicting.
